# Comment on “Identification of EMT-related high-risk stage II colorectal cancer and characterisation of metastasis-related genes”

**DOI:** 10.1038/s41416-020-01213-9

**Published:** 2020-12-14

**Authors:** Maurice B. Loughrey, Natalie C. Fisher, Aoife J. McCooey, Philip D. Dunne

**Affiliations:** 1 Department of Cellular Pathology, Belfast Health and Social Care Trust, Belfast, UK; 2 Centre for Public Health, Queen’s University Belfast, Belfast, UK; 3 The Patrick G. Johnston Centre for Cancer Research, Queen’s University Belfast, Belfast, UK

**Keywords:** Cancer microenvironment, Tumour biomarkers

We read with interest the publication by Wang et al.,^1^ which describes the development, testing and validation of an epithelial-to-mesenchymal transition (EMT)-related transcriptional classifier to identify patients with stage II colorectal cancer (CRC) at risk of disease relapse. Transcriptional characterisation of tumours classified as high risk using their approach, as anticipated, demonstrates a significant enrichment for gene sets associated with EMT. The authors conclude that their EMT signature identifies tumours with significantly elevated levels of epithelial transition, and that this transitional phenotype plays a more important role than the tumour microenvironment (TME) in driving the aggressive biology underpinning disease relapse. While we do not challenge the prognostic value of the classifier developed in this study, we do urge caution to the authors and across the field when utilising and interpreting such EMT-related signatures.

The process of EMT describes the transition of neoplastic epithelial cells towards a mesenchymal phenotype and/or transcriptional state. However, the classifier developed by Wang et al.^1^ may not reflect this transitional biology and is more likely to identify other features within the milieu of lineages that make up the TME. The use of genetic and transcriptionally perturbed cell lines and organoid models has enabled the development of highly refined and specific transcriptional signatures for research, including those used in the Molecular Signatures database (MSigDB). One such signature within the “Hallmark” collection is the EMT gene set and, while the development of this (and other EMT signatures) is based on experimentally validated data (as depicted in Fig. 1a), confusion arises through the imprecise interpretation of nomenclature when moving between molecular and histological phenotypes.^2^ This confounding issue is particularly evident when applying EMT-related classifiers in bulk tumour data where such signatures are a definitive surrogate marker of stroma/fibroblast content, as we have previously described (Fig. 1b).^3^ This is also clearly evident from the assessment of the cohorts used in the development/validation of the MSigDB EMT gene set, which employs differential signalling from tumour stroma and epithelium to define the EMT signature. From a biological sense, this is entirely appropriate, as these tumour samples are enriched for distinct mesenchymal and epithelial cell populations. This overlap between histology and transcriptomics is not an issue when EMT signatures are used to characterise the biological phenotype of purified cell lineages in vitro, where they give a robust measurement of epithelial or mesenchymal phenotype. However, when applied to bulk tumour transcriptional data, an EMT signature is significantly correlated with levels of cancer-associated fibroblasts (CAFs) and tumour stroma percentage, rather than an assessment of epithelial cells undergoing a phenotypic transition (Fig. 1b). While the prognostic value is true, confusion in nomenclature and biological descriptions attributed to a signature will inevitably promulgate uncertainty in the field and potentially drive inappropriate EMT-related research in epithelial cell lines that is unrelated to the true biology of these tumours.

**Fig. 1:**
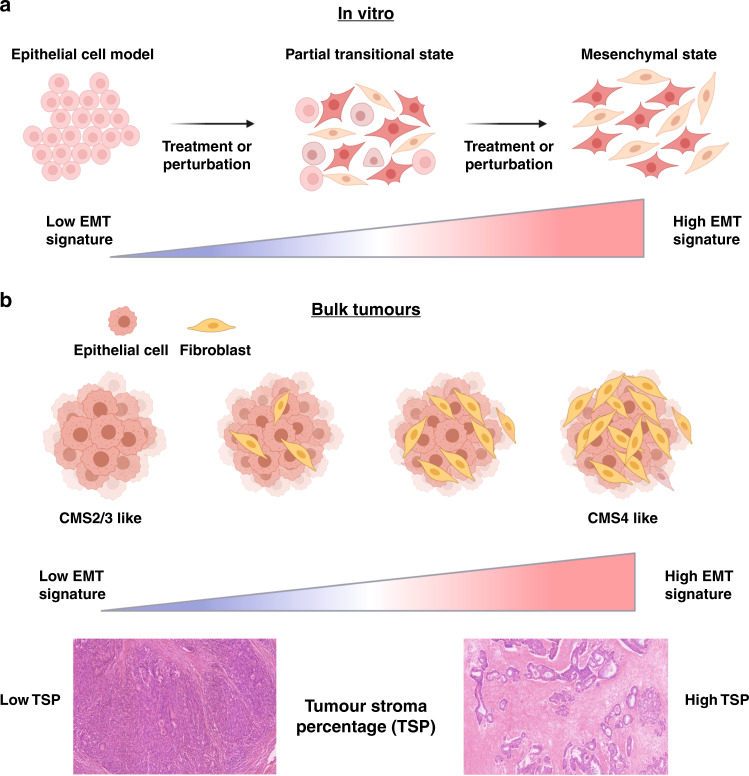
Comparison of epithelial-to-mesenchymal transition (EMT) signatures between in vitro cell line and bulk tumour profiling. **a** In vitro cell line models can be used to generate a “true” EMT signature. **b** When such EMT signatures are applied to bulk tumour samples they will correlate to the cancer-associated fibroblast content or tumour stroma percentage rather than the transitional biology they represent in cell line models. Image was produced using BioRender.

“True” EMT is likely too complex and dynamic a system to describe from bulk tumour data and such a level of granularity requires single-cell-level approaches, through methodologies such as single-cell RNA-sequencing^4^ or immunohistochemistry to examine in situ expression within tumour epithelial cells of specific EMT markers.^5^ Such EMT events are also most likely to be restricted to rare budding cells at the invasive front, rather than representing the majority of cells captured during bulk tumour profiling.^6^ In CRC, a series of molecular subtyping efforts over the past decade has culminated in the consensus molecular subtypes (CMS), which identified a poor-prognostic mesenchymal subtype (CMS4), characterised by increased levels of CAFs. These findings mirror a number of well-established histological approaches for prognostic classification, where the clinical value of assessing the TME is a proven and robust method for defining prognosis in stage II/III CRC.^7,8^ These stroma-rich tumours are reproducibly identifiable using such EMT signatures and/or gene sets and the stromal contribution to these transcriptional profiles is widely accepted.^9,10^

In summary, the classifier developed in this paper is certainly robust and offers potential clinical value. However, we propose that this is not based on EMT-related biology, as the chosen nomenclature would suggest, but rather CAF content, which would be immediately apparent upon histological review of the cohort. While the release of publicly available datasets in CRC has enabled researchers to perform important, paradigm-shifting transcriptomic characterisation studies, the absence of accompanying, ideally annotated, hematoxylin and eosin-stained images of the samples means that a key factor remains unseen, specifically the histological composition of the TME.

## Data Availability

Not applicable.
